# HomeCoRe for Telerehabilitation in Mild or Major Neurocognitive Disorders: A Study Protocol for a Randomized Controlled Trial

**DOI:** 10.3389/fneur.2021.752830

**Published:** 2021-12-23

**Authors:** Sara Bernini, Silvia Panzarasa, Elena Sinforiani, Silvana Quaglini, Stefano F. Cappa, Chiara Cerami, Cristina Tassorelli, Tomaso Vecchi, Sara Bottiroli

**Affiliations:** ^1^Dementia Research Center, Scientific Institute for Research, Hospitalization, and Healthcare (IRCCS) Mondino Foundation, Pavia, Italy; ^2^Department of Electrical, Computer and Biomedical Engineering, University of Pavia, Pavia, Italy; ^3^IUSS Cognitive Neuroscience (ICoN) Center, Scuola Universitaria di Studi Superiori IUSS, Pavia, Italy; ^4^Department of Brain and Behavioral Sciences, University of Pavia, Pavia, Italy; ^5^Headache Science and Neurorehabilitation Centre, Scientific Institute for Research, Hospitalization, and Healthcare (IRCCS) Mondino Foundation, Pavia, Italy; ^6^Cognitive Psychology Research Center, Scientific Institute for Research, Hospitalization, and Healthcare (IRCCS) Mondino Foundation, Pavia, Italy; ^7^Faculty of Law, Giustino Fortunato University, Benevento, Italy

**Keywords:** neurocognitive disorder, dementia, computer-based telerehabilitation, cognitive training, mild cognitive impairment, cognitive rehabilitation

## Abstract

**Background:** Given the limited effectiveness of pharmacological treatments for cognitive decline, non-pharmacological interventions have gained increasing attention. Evidence exists on the effectiveness of cognitive rehabilitation in preventing elderly subjects at risk of cognitive decline and in reducing the progression of functional disability in cognitively impaired individuals. In recent years, telerehabilitation has enabled a broader application of cognitive rehabilitation programs. The purpose of this study is to test a computer-based intervention administered according to two different modalities (at the hospital and at home) using the tools CoRe and HomeCoRe, respectively, in participants with Mild or Major Neurocognitive Disorders.

**Methods:** Non-inferiority, single-blind randomized controlled trial where 40 participants with Mild or Major Neurocognitive Disorders will be assigned to the intervention group who will receive cognitive telerehabilitation through HomeCoRe or to the control group who will receive in-person cognitive intervention through CoRe, with the therapist administering the same computer-based exercises. The rehabilitative program will last 6 weeks, with 3 sessions/week, each lasting ~45 min. All the participants will be evaluated on an exhaustive neuropsychological battery before (T0) and after (T1) the intervention; follow-up visits will be scheduled after 6 (T2) and 12 months (T3).

**Discussion:** The results of this study will inform about the comparability (non-inferiority trial) of HomeCoRe with CoRe. Their equivalence would support the use of HomeCoRe for at distance treatment, favoring the continuity of care.

**Ethics and Dissemination:** This study has been approved by the Local Ethics Committee and registered in https://clinicaltrials.gov (NCT04889560). The dissemination plan includes the scientific community through publication in open-access peer-reviewed scientific journals and presentations at national and international conferences.

**Trial Registration:** Clinicaltrials.gov https://clinicaltrials.gov/ct2/show/NCT04889560 (registration date: May 17, 2021).

## Introduction

In the light of the limited efficacy of pharmacological therapies for cognitive decline, the management of modifiable risk factors affecting age-associated cognitive decline and risk of dementia is attracting an increasing interest (1, 2). There is some evidence that early cognitive interventions may be effective in individuals in predementia phases (3, 4). Mild cognitive impairment (MCI) (3) or Mild Neurocognitive Disorder according to the *Diagnostic and Statistical Manual of Mental Disorders-5* (DSM-5) (5) is defined as a transitional status between normal aging and possible development of early dementia. It is characterized by subjective cognitive complaints and objective cognitive decline greater than expected for age and education levels of an individual, but not interfering with activities of daily life. Dementia (i.e., Major Neurocognitive Disorder, according to DSM-5), defined according to severity level, is characterized by multidomain cognitive deficits resulting in a significant interference with independence in everyday activities (5).

In this field of research, previous studies demonstrated the effectiveness of cognitive training programs in patients in the early stage (i.e., MCI and mild dementia) of cognitive decline (6, 7). Traditionally, cognitive interventions consist of in-person sessions usually administered in the hospital setting under supervision by a therapist using paper-and-pencil techniques or technology-based solutions. In particular, the use of technology promotes the development of *ad-hoc* (i.e., user tailored) cognitive rehabilitation tools, allowing to overcome the limits associated to paper-and-pencil techniques. Recent advances in technologies allow for a new and innovative implementation of treatments [i.e., telerehabilitation (TR)], which can be easily diffused on large scale and guarantee a continuum of care at distance (4, 8).

Despite the interesting potentialities of TR, several issues are slowing its integration into the clinical routine. A major issue is the poor technological skills of older adults, which may result in difficulties in managing technological devices autonomously (9). Therefore, platforms should be accessible and user-friendly; duration and frequency of rehabilitation activities should vary according to characteristics of the patients (10); therapists should monitor adherence to treatment and outcome of the rehabilitation process remotely (11). There is some evidence (12–17) exploring the usability and acceptability of Information and Communication Technologies (ICTs) in elderly care including participants with dementia or MCI and giving some recommendations for designing interfaces for this kind of users. In general, it resulted that these systems were enjoyable and feasible for participants even if usability not always was high.

However, randomized controlled trials (RCTs) investigating TR efficacy compared to the traditional in-person approaches are still scanty (4). Recently, this topic has gained growing interest, due to the challenges faced by the healthcare systems during coronavirus disease 2019 (COVID-19) pandemic (18–22).

During the past years, we implemented the software CoRe for in-person cognitive training in the hospital setting supervised by a trainer (23, 24). CoRe has been successfully tested in terms of usability and immediate and long-term effectiveness in participants with early cognitive decline (25–27). In light of the improvement in telemedicine approaches and in view of the willingness of treated participants and caregivers to start/continue CoRe program at distance (28), we have developed the “home” version of CoRe (i.e., HomeCoRe) supporting cognitive intervention remotely (29) with the assistance of a family caregiver.

This longitudinal RCT study, thus, aims to evaluate and compare the effectiveness of HomeCoRe and CoRe programs in participants with MCI or mild dementia. Our hypothesis is that cognitive TR delivered via HomeCoRe provides benefits that are comparable to the in-person version of the program on cognitive and behavioral functioning and on additional participant-centered outcomes.

We are currently performing a small-scale usability test on the HomeCoRe system with encouraging results. The first six participants who completed the usability test considered the HomeCoRe system as an innovative and original tool that they integrated smoothly and positively in their daily life routine. These participants are providing us crucial feedback to improve the system usability such as the need of extra time for performing exercises. Based on the feedback received, HomeCoRe is undergoing a refinement process that will lead to the final version to be used in the RCT.

## Methods and Analysis

### Study Design

**Figure 1:**
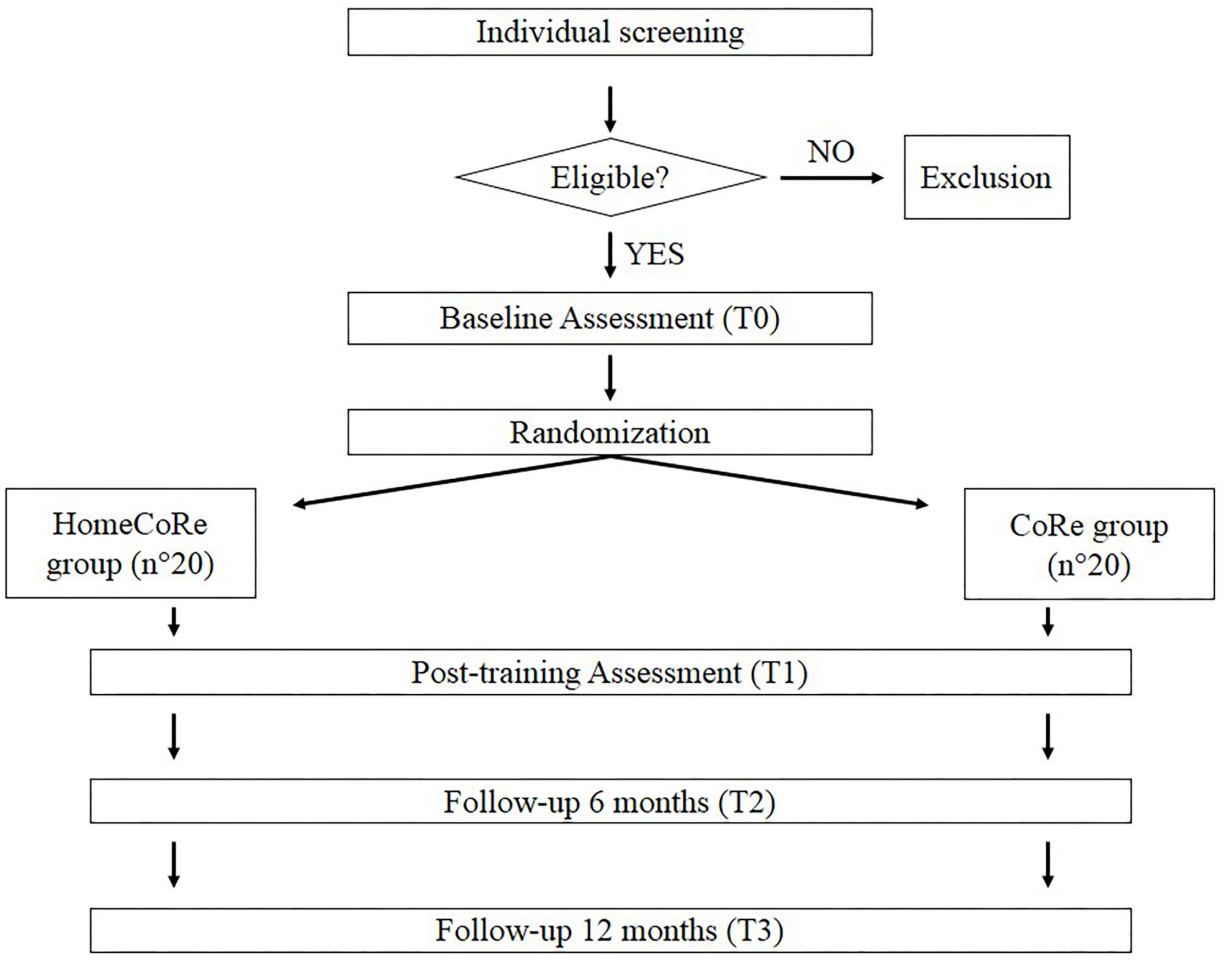
The Consolidated Standards of Reporting Trials (CONSORT) flowchart for enrollment and randomization.

This study is a prospective single-blind randomized controlled non-inferiority trial. The Consolidated Standards of Reporting Trials (CONSORT) flowchart for enrollment and randomization is shown in Figure 1. After recruitment, participants will be contacted and will undergo in-person baseline assessment (T0) using the below-listed tests (see evaluation of the participants section and Table 1). Participants who meet the inclusion criteria will be enrolled and randomized to one of two groups: HomeCoRe and CoRe. For both the groups, the intervention will consist of a 6-week program (3 sessions/week, each lasting ~45 min). Follow-up in-person neuropsychological assessments will be scheduled at the end of the rehabilitation program (T1) and after 6 (T2) and 12 months (T3).

**Table 1:** Evaluation battery across testing sessions.

	**T0**	**T1**	**T2**	**T3**
**Neuropsycological assessment**				
***Global cognition***				
Mini-Mental State Examination (MMSE)	x^*^	x^*^	x	x
Montreal Cognitive Assessment (MoCA)	x	x	x	x
***Episodic long-term memory***				
Logical Memory Test immediate and delayed recall	x	x	x	x
Rey's 15 words test immediate and delayed recall	x	x	x	x
Rey Complex Figure delayed recall	x	x	x	x
***Logical-executive functions***				
Rey Complex Figure copy	x	x	x	x
Raven's Matrices 1947	x	x	x	x
Frontal Assessment Battery (FAB)	x	x	x	x
Semantic fluency	x	x	x	x
Phonological fluency (FAS)	x	x	x	x
***Working memory***				
Verbal Span	x	x	x	x
Digit Span	x	x	x	x
Corsi's block-tapping test span	x	x	x	x
***Attention/processing speed***				
Attentive matrices	x	x	x	x
Trail Making Test A and B (TMT)	x	x	x	x
**Questionnaires and scales**				
***Functional level***				
Activities of Daily Living (ADL)	x			x
Instrumental Activities of Daily Living (IADL)	x			x
***Depressive symptoms***				
Beck Depression Inventory (BDI)	x	x	x	x
***Health status***				
36-Item Short Form Health Survey questionnaire (SF-36)	x	x	x	x
***Cognitive reserve***				
Cognitive Reserve Index questionnaire (CRIq)	x			
***Caregiver distress***				
Caregiver Burden Inventory (CBI)^+^	x	x		
**Participant-centered outcomes**				
***Impression of symptom change***				
Patient Global Impression of Change (PGIC)		x		
***Treatment adherence***				
Number of sessions carried out		x		

*T0, baseline assessment; T1, post-intervention assessment; T2, 6-month follow-up; T3, 12-month follow-up*.

* *Is used for the primary outcome,*

+ *is used only for caregivers of participants with Major Neurocognitive Disorder*.

#### Data Collection

Assessments will take place at the Scientific Institute for Research, Hospitalization, and Healthcare (IRCCS) Mondino Foundation (Pavia, Italy). Neuropsychologists carrying out evaluations will receive appropriate instruction and guidance regarding all the assessment procedures and outcome parameters. Reminders (e.g., written reminder, phone calls, and email message) for each visit will be given to all the participants. Research staff collecting data will be blind to group allocation. Not all the outcome measures will be administered at each time point (Table 1).

#### Data Management

Study data will be recorded in the database in processes compliant with the General Data Protection Regulation (GDPR). All the participants will be registered with an identification code. The database will be kept updated to reflect the status of the participant at each stage during the course of this study. The collected data, after scientific publication, will be deposited in dedicated repositories (e.g., Zenodo) according to the good practice of data sharing.

### Participants and Eligibility Criteria

Participants will be recruited from the Dementia Research Center outpatient services and Neurorehabilitation Unit of the IRCCS Mondino Foundation (Pavia, Italy) and screened for eligibility criteria through a clinician evaluation made by an expert neurologist.

The inclusion criteria for participants will be:

- Diagnosis of Mild or Major Neurocognitive Disorders based on the DSM-5 (5)
- Aged between 60 and 85 years
- Years of education ≥ 5
- Clinical Dementia Rating (CDR) (30) score = 0.5–1.

The exclusion criteria will be:

- Mini-Mental State Examination (MMSE) score <20
- Presence of cognitive impairment secondary to an acute or general medical disorder (e.g., brain trauma or tumor)
- Presence of severe neuropsychiatric conditions (e.g., mood and behavioral disorders)
- Presence of severe sensory disorder (e.g., deafness or blindness) or motor impairment that prevent trunk control and/or sitting position
- Current cognitive treatments
- Lack of family support for participants with Major Neurocognitive Disorder

Medication intake for dementia and/or past cognitive rehabilitation treatments will be not considered as exclusion criteria given that these factors are not expected to affect the outcome of this study. Any pharmacological treatment ongoing must be stable across the entire period of this study protocol.

### Evaluation of the Participants

Table 1 lists the evaluation battery (neuropsychological assessment, questionnaires and scale, and participant-centered outcomes) across testing sessions. Each evaluating session would last about 90 min per participant and will be carried out in a hospital setting.

#### Neuropsychological Assessment

The cognitive assessment, performed by using neuropsychological tests standardized for the Italian population, will evaluate the following cognitive domains:

- Global cognition:
  ° Mini-Mental State Examination (31)
  ° Montreal Cognitive Assessment (31, 32)
- Episodic long-term memory:
  ° Logical Memory Test for immediate and delayed recall (33, 34)
  ° Rey's 15 words test for immediate and delayed recall (35)
  ° Rey Complex Figure delayed recall (36)
- Logical-executive functions:
  ° Raven's Matrices 1947 (35)
  ° Frontal Assessment Battery (37)
  ° Semantic fluency (33)
  ° Phonological fluency (FAS) (35)
  ° Rey Complex Figure copy (36)
- Working memory:
  ° Verbal Span (34)
  ° Digit Span (34)
  ° Corsi block-tapping test span (34)
- Attention/processing speed:
  - ° Attentive Matrices (34)
  - ° Trail Making Test A and B (38).

Parallel forms (i.e., alternative versions using similar material) will be applied for follow-up visits when available in order to avoid the learning effect. All the test scores will be corrected for age, sex, and education by using appropriate correction grids and compared with the values available for the Italian population.

#### Questionnaires and Scales

Additionally, we will administer questionnaires and scales reported below to evaluate the following aspects:

- Functional level:
  ° Activities of Daily Living (39)
  ° Instrumental Activities of Daily Living (39)
- Depressive symptoms:
  ° Beck Depression Inventory (40)
- Health status:
  ° 36-item Short Form Health Survey questionnaire (41)
- Cognitive reserve:
  ° Cognitive Reserve Index questionnaire (42)
- Caregiver distress:
  ° Caregiver Burden Inventory (43) only for caregivers of participants with Major Neurocognitive Disorder.

#### Participant-Centered Outcomes

In order to assess subjective evaluation of TR success, we will evaluate the following aspects:

- Impression of symptom change:
  ° Patient Global Impression of Change (44)
- Treatment adherence:
  ° Number of sessions carried out.

### Randomization and Stratification

After baseline assessment, we will generate random numbers through the use of a computer algorithm (https://www.random.org/) from a uniform distribution in the range 0–1, dividing the range in two equal intervals and assigning each participant to the group corresponding to the sampled number (1:1 ratio), within strata defined by diagnosis (Mild or Major Neurocognitive Disorders). Neuropsychologists carrying out cognitive evaluations will be blinded to group allocation.

### Cognitive Rehabilitation Programs

**Figure 2:**
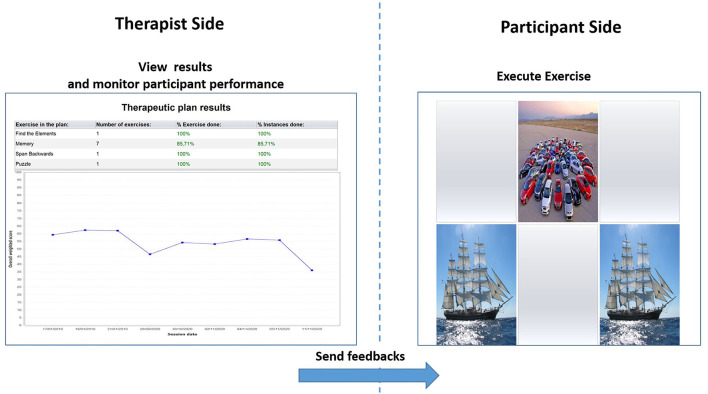
Therapist interface for monitoring performances of the participant in terms of overall Weighted Score (left) and interface of the participant for the execution of exercises (right).

Both the CoRe and HomeCoRe are research software tools developed within a long-lasting collaboration between clinicians from the IRCCS Mondino and bioengineers from the University of Pavia. At the moment, the tools are limited to Italian speaking participants. The tools allow a participant-tailored intervention aimed at stimulating several cognitive abilities (e.g., logical-executive functions, attention/processing speed, working memory, and episodic memory) through a series of sessions of exercises (see Table 2 for details). Their use is time-saving for the therapists, as they are ready to use and do not require a continuous manual setting of exercises for each training session. This is because, once the therapist has remotely set up the treatment plan, exercises take place in an adaptive mode across all the sessions. In particular, during their dynamic generation, performance data of an individual participant are analyzed in order to set the appropriate difficulty level. Performance data of the participants refer to the response accuracy normalized according to the number of aids that the participant required to solve the task. Furthermore, for each exercise and each level, thresholds are defined to allow difficulty levels to progressively increase in order to stimulate neural plasticity (6, 45, 46). In addition, the system calculates an overall “Weighted Score” (WS), taking into account the correctness of the answers, the execution time, and the difficulty of the exercises. The WS informs the therapist about each performance of the participant in a single value. Hence, WS represents a useful and advantageous index that can be used to assess both the overall outcome of a training session and the global trend of the rehabilitation (see Figure 2).

**Table 2:** CoRe/HomeCoRe description of tasks, reporting involved cognitive skills.

**Tasks**	**Description**	**Main involved skills**
***Learning of word pairs***	Pairs of words are shown on the screen, the participant is asked to rewrite the second word of the pair when it is shown	Long-term memory abilities; learning and re-enactment strategies; visual imagery
***Word categorization***	Words belonging to different categories are presented on the screen, the patient is asked to rewrite them in any order but respecting the corresponding category	Long-term memory abilities; learning and re-enactment strategies; visual imagery; categorical thinking
***Puzzle***	The participant is asked to put together the pieces of a figure to recompose it	Visuo-spatial long-term memory; visual imagery; mental representation and planning
***Span backward***	The participant is asked to write the numbers in a reverse order compared to what previously heard	Verbal working memory; processing-speed
***Memory***	After a study phase in which all cards are shown face up, they are faced down. The participant is asked to recall and match all equal cards in the least number of tries, by turning over pairs of cards one by one	Long-term memory abilities; visuo-spatial abilities
***Visuospatial matrices***	The participant receives a sequence of spatial information (e.g., right, left, up, down) and then he/she is asked to store it and reproduce in the correct order on a grid	Working memory; visuo-spatial abilities; processing-speed
***Logical sequences***	A sequence of images is shown, the participant is asked to select, among several options, the one that completes the series	Non-verbal reasoning; mental problem solving; decision making
***Image and sound***	The participant is asked to evaluate right or wrong matching between visual (image size) and auditory (sound volume) stimuli	Inhibitory control; processing-speed; working memory
***Sentence recomposing***	The participant is asked to put scrambled words in the correct order to form a full sentence	Mental and verbal planning; conceptual abstraction abilities
***Story recomposing***	The participant is asked to reorganize a scrambled sequence of images in the right chronological order to form a short story	Planning of activities: problem solving; temporal sequencing; visual attention
***Recognition exercise***	The participant is asked to identify and select specific items within a matrix of random elements (letters or numbers)	Sustained and selective attention; visuo-spatial scanning; processing-speed

### CoRe/HomeCoRe Software Architecture

Both the CoRe and HomeCoRe require a personal computer equipped with a touch screen. HomeCoRe is installed on a laptop (password protected and encrypted) that is supplied to participants by the therapist, while CoRe is installed on a desktop PC located in the hospital setting. Both the HomeCoRe and CoRe will be installed on the personal computer by an expert engineer and under the supervision of the Information Technology (IT) department—IRCCS Mondino. CoRe, being an in-person treatment, will be then performed under therapist monitoring; HomeCoRe, being home based, will/could be performed under caregiver monitoring. In particular, before the beginning of HomeCoRe treatment, participants and possible caregivers will be trained together at the hospital on the use of the rehabilitation tool at home. This is in order to account for possible differences in baseline technological skills. Then, during the training sessions, participants, with the possible support of their caregivers, will go through each exercise of the treatment until they feel familiar with the use of the device. During the rehabilitative program at home, remote technical support will be available when requested. To this aim, participant will be provided with the support team contacts. The treatment sessions, both in the CoRe and HomeCore, can be paused in case of fatigue of the participant and resumed at a later time.

**Figure 3:**
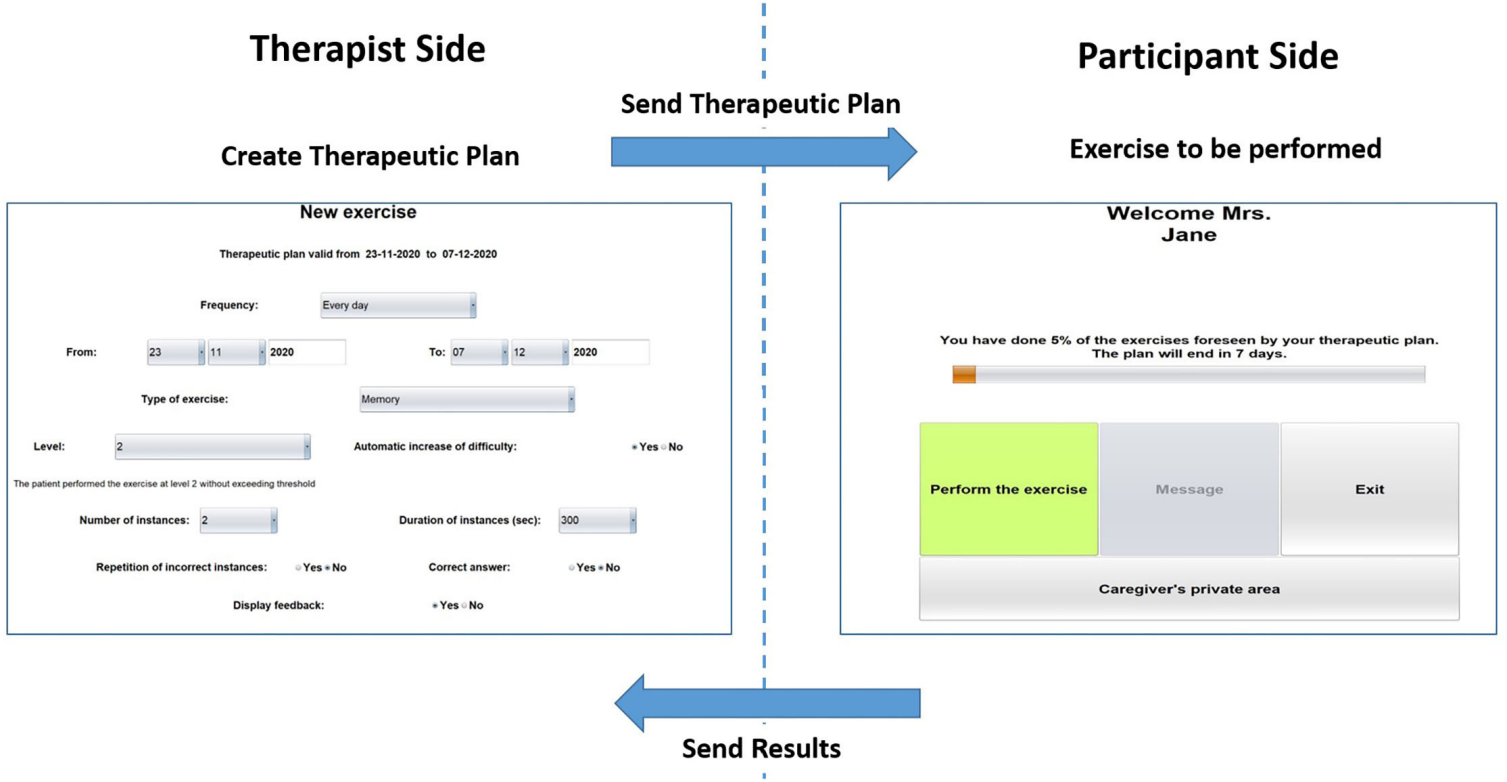
Home page of the therapist side of the interface for setting the requirements for the exercise plan (left) and home page of the participant/caregiver side of the interface (right) for HomeCoRe.

Differently from CoRe, HomeCoRe architecture includes two main components, namely, therapist side and participant/caregiver side and a communication system (HomeCoRe server). The therapist-side dashboard allows remotely setting and monitoring all the parameters of the treatment plan (e.g., frequency and duration of the plan, type of exercises, difficulty level). The interface of the participant/caregiver is very simple and it allows to view/execute the exercises of the day and to send the results to the therapist (see Figure 3).

The HomeCoRe system can be used online or offline in the case that the internet connection of the participant is not available. In the online mode, the communication between the therapist side and participant side takes place automatically through a dedicated communication protocol managed by the HomeCoRe server, while in the offline modality, some manual operations are required for loading the therapeutic plan offline and save result report on an external memory support (e.g., USB key or hard disk). In any case, the communication with the therapist is asynchronous.

### Outcome Measures

As the primary outcome measure, we will consider the change in global cognitive functioning, measured using the MMSE at T1 compared to T0. Secondary outcome measures will be longitudinal changes in all the neuropsychological tests, questionnaires and scales (T1, T2, and T3 vs. T0 when applicable according to Table 1). Secondary outcome measures will include also participant-centered outcomes to assess those aspects that are most important for the participants and the subjective evaluation of intervention success at T1.

### Statistical Analysis

#### Sample Size Calculation

Sample size has been estimated based on previous evidence in the literature (47). Since this is a non-inferiority study, we will consider as margin *d* a value of two points difference (T1 vs. T0) at the MMSE between the two groups. We predict to obtain a mean difference between HomeCoRe and CoRe groups of about one point at the MMSE with an SD of 1. Considering an alpha significance of 0.05 and a power of 0.9, the sample size for a non-inferiority study is 18 participants per group, for a total of 36 participants. It is planned to enroll a total of 40 participants in order to account for possible dropouts. If dropout rates between T0 and T1 will be higher than expected, extra participants will be recruited. The sample size for non-inferiority studies was calculated using R 4.0.2 software, SampleSize4ClinicalTrials package.

#### Planned Analysis

Statistical analysis on outcome measures will be conducted using the SPSS software (see Supplementary Materials for planned analysis). A normality test will be used to assess the distribution of all the outcome measures. Baseline differences between groups will then be tested using the independent samples *t*-test for parametric data and the Mann–Whitney *U*-test for non-parametric data. Within-group statistical tests will be performed for both the CoRe and HomeCoRe groups to look for significant changes in primary and secondary outcome measures over time. Between-group tests will be performed to look for differences in primary and secondary outcome measures between HomeCoRe and CoRe participants. Possible between-group differences in demographic and clinical characteristics (e.g., age, sex, years of education, diagnosis, and cognitive reserve) and in T0 scores in primary and secondary outcome measures will be considered as possible confounders and will be treated as covariates in the analysis. *p* ≤ 0.05, corrected for multiple comparisons, if appropriate, will be considered as statistically significant.

### Ethical Issues

This study will involve human participants, cognitive rehabilitation interventions, data collection, elaboration, and abstraction used for the evaluation of the two therapeutic options. In addition to ethical approval, all the procedures and the data managed have been approved by the Data Protection Officer of the IRCCS Mondino who guarantees compliance to the GDPR. The information provided when presenting the informed consent to the participants will be given in a language appropriate to the individual level of understanding. Participants will also be encouraged to ask questions before signing the informed consent.

To the best of our knowledge, HomeCoRe should not have any potential negative impact on the participant. The investigator will communicate any possible, unforeseen, and adverse event to the Ministry of Health. With respect to payment policies for participants, the amount of compensation and the method and timing of disbursement must be consistent with the laws, regulations, and guidelines of the region in which this study is conducted and must not improperly influence a decision of the participant to participate. This study is a no-profit study and, in Italy, the national legislation refers that it is forbidden to offer or request any kind of financial benefit for the participation in a clinical experimental trial.

Since participants are expected to interact with a rehabilitation tool (the HomeCoRe application), one possible issue could be frustration in case of lack of ability to cope with that technology. However, this risk will be mitigated, before the beginning of HomeCoRe treatment, thanks to specific training sessions on the use of this application that will be delivered to participants (and possible caregivers) (see CoRe/HomeCoRe software architecture section). Moreover, the interface fully complied with the guidelines for human–computer interaction, to make the user interface as easy as possible.

## Discussion

Due to the increase of the aging population, we are witnessing a steady increase in the number of older adults at risk of developing cognitive decline with a consequent increase of economic burden on healthcare. Therefore, the WHO Global Action Plan on the Public Health Response to Dementia 2017–2025 recommends taking global action against cognitive decline and dementia, encouraging governments worldwide to focus on prevention and improving healthcare services (48). Telemedicine is defined as an interface in a virtual patient–clinician relationship to provide primary and secondary care by adopting innovative solutions reaching larger groups of participants (49). Telemedicine can be considered as an adaptation of the healthcare model based on in-person interaction, according to the characteristics and needs of the participants (50). In particular, TR is a telemedicine subfield aimed at providing rehabilitation at a distance (51). TR provides benefits for the healthcare system, patients, and caregivers in terms of cost-effectiveness and feasibility for large-scale implementations (52–54). It represents a replacement for in-person treatment or its continuation, favoring equitable access to care not only for older patients with dementia or physical disabilities, but also for subjects of working age or living in geographically remote areas in predementia phases. Hence, TR is a unique opportunity in the field of cognitive rehabilitation to guarantee constancy and continuity to cognitive training programs.

The results of this trial will inform about the comparability of HomeCoRe with CoRe system. In case they will result equivalent, such a finding would support the use of HomeCoRe in the treatment of patient at distance, with the consequent multiple positive impacts mentioned above. In this framework, HomeCoRe could be incorporated into clinical routine practices as a complementary non-pharmacological therapy to contrast cognitive impairment and dementia. In case HomeCoRe will prove less effective than CoRe, it would lead to the conception of telerehabilitation as a compromise that must be made under particular conditions such as in case of emergency [i.e. coronavirus disease 2019 (COVID-19) pandemic] or personal needs (e.g., travel difficulties).

### Strengths and Limitations

This RCT will allow to implement and assess the effectiveness of a TR tool targeting participants with cognitive decline. HomeCoRe aims to provide participant-tailored cognitive intervention directly at home, also when needed to extend the duration of cognitive programs started in the hospital setting and to reduce the dropout rate. The availability of effective and feasible TR modalities will address the paucity of healthcare personnel dedicated to cognitive rehabilitation within the neuropsychology services, thus increasing the offer to a wider population. It will also provide a modality to ensure care continuity also during COVID-19 pandemic crises.

This study has some limitations that need to be acknowledged. In particular, participants with scanty computer familiarity and without a compliant caregiver could be excluded by the use of TR, representing a selection bias for this kind of intervention (55). However, there is also evidence about the possibility of using telemedicine devices in participants with early cognitive impairment living alone. It seems that compliance of the participants depends on the level of monitoring remotely received (56). In addition, it is important to consider that user-friendly developed TR tools can produce benefits in participants and also caregivers (57).

## Ethics Statement

This study has been approved by the Local Ethics Committees (IRCCS San Matteo Hospital, Pavia) and will be conducted in accordance with the Declaration of Helsinki and reported according to the CONSORT guidelines (58, 59). The trial was registered at clinicaltrials.gov (NCT number: NCT03486704). All the participants will be made fully aware of the aims of this study and a written informed consent will be obtained from all the subjects.

## Author Contributions

SBe and SBo developed the original concept of the study, drafted the original protocol, and wrote the manuscript. SBe, SBo, SQ, SP, and ES developed the design, the methodology, and the analysis plan. SQ, SP, ES, SFC, CC, TV, and CT reviewed and commented on drafts of the protocol and study. All authors read and approved the final manuscript.

## Funding

This study was supported by a grant from the Italian Ministry of Health (Ricerca Corrente 2020 and Ricerca Corrente 2021).

## Conflict of Interest

The authors declare that the research will be conducted in the absence of any commercial or financial relationships that could be construed as a potential conflict of interest.

## Publisher's Note

All claims expressed in this article are solely those of the authors and do not necessarily represent those of their affiliated organizations, or those of the publisher, the editors and the reviewers. Any product that may be evaluated in this article, or claim that may be made by its manufacturer, is not guaranteed or endorsed by the publisher.
