# Supplementary material for: HomeCoRe for Telerehabilitation in Mild or Major Neurocognitive Disorders: A Study Protocol for a Randomized Controlled Trial
Source: Front Neurol. 2021 Dec 23;12:752830. doi: 10.3389/fneur.2021.752830 (PMC8733654; doi:10.3389/fneur.2021.752830)
Supplement: Supplementary file 1 [file Table_1.DOCX]

Supplementary material 1. *Participant characteristics (means ± standard Deviations) as a function of group.*

|  | **HomeCore**  (n=) | **CoRe**  (n=) | | | **Independent T-test/Mann-Whitney Test** | | |
| --- | --- | --- | --- | --- | --- | --- | --- |
| Age |  | |  |  | |  |  |
| Years of education |  | |  |  | |  |  |
| % Female |  | |  |  | |  |  |
| % Mild Neurocognitive Disorder |  | |  |  | |  |  |
| Caregiver support* |  | |  |  | |  |  |
| Cognitive reserve |  | |  |  | |  |  |
| *Note*: *only for participants in the HomeCoRe group. | | | | | | | |

Supplementary material 2. *Mean values ± standard deviations recorded for cognitive evaluations at T0, T1, T2, and T3 as a function of group*.

|  |  | | | |  | |  |  |  |  |  |  |
| --- | --- | --- | --- | --- | --- | --- | --- | --- | --- | --- | --- | --- |
|  | **HomeCoRe** | | | | | **CoRe** | | | | | |  |
|  | T0 | T1 | T2 | T3 | | T0 | | T1 | T2 | T3 |  |  |
| Global cognition |  |  |  |  | |  | |  |  |  |  |  |
| Episodic long-term memory |  |  |  |  | |  | |  |  |  |  |  |
| Executive functions |  |  |  |  | |  | |  |  |  |  |  |
| Working memory |  |  |  |  | |  | |  |  |  |  |  |
| Attention/processing speed |  |  |  |  | |  | |  |  |  |  |  |
| Functional level |  |  |  |  | |  | |  |  |  |  |  |
| Depressive symptoms |  |  |  |  | |  | |  |  |  |  |  |
| Health status |  |  |  |  | |  | |  |  |  |  |  |
| Caregiver distress* |  |  |  |  | |  | |  |  |  |  |  |
| Impression of symptom change |  |  |  |  | |  | |  |  |  |  |  |
| Treatment adherence |  |  |  |  | |  | |  |  |  |  |  |
| Note. * only for caregivers of participants with Major Neurocognitive Disorder. | | | | | | | | | | |  |  |

Supplementary material 3. *Intra-group comparisons for the HomeCoRe group*.

| **HomeCoRe** | **T0 vs T1** | | | **T0 vs T2** | | | | **T0 vs T3** | | |
| --- | --- | --- | --- | --- | --- | --- | --- | --- | --- | --- |
|  | Z | W(p) Intra-g | d |  | Z | W(p) Intra-g | d | Z | W(p) Intra-g | d |
| Global cognition |  |  |  |  |  |  |  |  |  |  |
| Episodic long-term memory |  |  |  |  |  |  |  |  |  |  |
| Logical-executive functions |  |  |  |  |  |  |  |  |  |  |
| Working memory |  |  |  |  |  |  |  |  |  |  |
| Attention/processing speed |  |  |  |  |  |  |  |  |  |  |
| Functional level |  |  |  |  |  |  |  |  |  |  |
| Depressive symptoms |  |  |  |  |  |  |  |  |  |  |
| Health status |  |  |  |  |  |  |  |  |  |  |
| Caregiver distress* |  |  |  |  |  |  |  |  |  |  |
| Impression of symptom change |  |  |  |  |  |  |  |  |  |  |
| Treatment adherence |  |  |  |  |  |  |  |  |  |  |
| *Note*: W = Wilcoxon signed-rank test; d = Cohen’s d effect size; * only for caregivers of participants with Major Neurocognitive Disorder. | | | | | | | | | | |

Supplementary material 4. *Intra-group comparisons for the CoRe group*.

| **CoRe** | **T0 vs T1** | | | **T0 vs T2** | | | | **T0 vs T3** | | |
| --- | --- | --- | --- | --- | --- | --- | --- | --- | --- | --- |
|  | Z | W(p) Intra-g | d |  | Z | W(p) Intra-g | d | Z | W(p) Intra-g | d |
| Global cognition |  |  |  |  |  |  |  |  |  |  |
| Episodic long-term memory |  |  |  |  |  |  |  |  |  |  |
| Logical-executive functions |  |  |  |  |  |  |  |  |  |  |
| Working memory |  |  |  |  |  |  |  |  |  |  |
| Attention/processing speed |  |  |  |  |  |  |  |  |  |  |
| Functional level |  |  |  |  |  |  |  |  |  |  |
| Depressive symptoms |  |  |  |  |  |  |  |  |  |  |
| Health status |  |  |  |  |  |  |  |  |  |  |
| Caregiver distress* |  |  |  |  |  |  |  |  |  |  |
| Impression of symptom change |  |  |  |  |  |  |  |  |  |  |
| Treatment adherence |  |  |  |  |  |  |  |  |  |  |
| *Note*: W = Wilcoxon signed-rank test; d = Cohen’s d effect size; * only for caregivers of participants with Major Neurocognitive Disorder. | | | | | | | | | | |
